# Treatment with 1,25(OH)_2_D_3_induced HDAC2 expression and reduced NF-κB p65 expression in a rat model of OVA-induced asthma

**DOI:** 10.1590/1414-431X20154271

**Published:** 2015-04-28

**Authors:** Y. Zhou, G.F. Wang, L. Yang, F. Liu, J.Q. Kang, R.L. Wang, W. Gu, C.Y. Wang

**Affiliations:** Department of Gerontology Medicine, Xinhua Hospital, Shanghai Jiatong University School of Medicine, Shanghai, China

**Keywords:** 1,25-Dihydroxyvitamin D3, Asthma, Acetylation, Deacetylation, Histone deacetylase 2, NF-κB

## Abstract

Recent evidence indicates that a deficiency of 1,25-dihydroxyvitamin D_3_ (1,25[OH]_2_D_3_) may influence asthma pathogenesis; however, its roles in regulating specific molecular transcription mechanisms remain unclear. We aimed to investigate the effect of 1,25(OH)_2_D_3_ on the expression and enzyme activity of histone deacetylase 2 (HDAC2) and its synergistic effects with dexamethasone (Dx) in the inhibition of inflammatory cytokine secretion in a rat asthma model. Healthy Wistar rats were randomly divided into 6 groups: control, asthma, 1,25(OH)_2_D_3_ pretreatment, 1,25(OH)_2_D_3_ treatment, Dx treatment, and Dx and 1,25(OH)_2_D_3_ treatment. Pulmonary inflammation was induced by ovalbumin (OVA) sensitization and challenge (OVA/OVA). Inflammatory cells and cytokines in the bronchoalveolar lavage (BAL) fluid and histological changes in lung tissue were examined. Nuclear factor kappa B (NF-κB) p65 and HDAC2 expression levels were assessed with Western blot analyses and quantitative reverse-transcriptase polymerase chain reaction (qRT-PCR). Enzyme activity measurements and immunohistochemical detection of HDAC2 were also performed. Our data demonstrated that 1,25(OH)_2_D_3_ reduced the airway inflammatory response and the level of inflammatory cytokines in BAL. Although NF-κB p65 expression was attenuated in the pretreatment and treatment groups, the expression and enzyme activity of HDAC2 were increased. In addition, 1,25(OH)_2_D_3_ and Dx had synergistic effects on the suppression of total cell infusion, cytokine release, and NF-κB p65 expression, and they also increased HDAC2 expression and activity in OVA/OVA rats. Collectively, our results indicated that 1,25(OH)_2_D_3_might be useful as a novel HDAC2 activator in the treatment of asthma.

## Introduction

Allergic asthma is a chronic inflammatory disease characterized by increased bronchial responsiveness, constriction, and mucus hypersecretion in the bronchial walls (1). The symptoms of most patients with asthma are well controlled with low doses of inhaled corticosteroids. The major mechanism of action of corticosteroids in the suppression of inflammation in asthma is to switch off multiple activated inflammatory genes encoding cytokines, chemokines, adhesion molecules, inflammatory enzymes, and receptors (2). Nuclear factor kappa B (NF-κB) is a ubiquitous transcription factor involved in the proinflammatory response and is activated in asthma, especially severe and steroid-resistant subtypes (3,4). NF-κB consists of homo- or heterodimers of different subunits, such as p50, p52, p65/RelA, RelB, and c-Rel, with p65/RelA and p50 being the most common and well studied (5,6). The p65 protein is a key active component of NF-κB. At lower concentrations, glucocorticoids (GCs) reduce the inflammatory gene transcription induced by NF-κB or activator protein 1 (AP-1) via an association between these factors and GC receptor (GR) (7). Although the mechanisms are not fully understood, recent studies have shown that changes in the epigenetic profile regulate the expression and activation of NF-κB to mediate steroid action in chronic obstructive pulmonary disease (COPD) and asthma (8,9).

Histone acetylation/deacetylation is an epigenetic event that plays an important role in inflammation (10,11). Histone acetyltransferase-mediated acetylation of specific lysine residues on the N-terminal tail of core histones results in DNA uncoiling and increased accessibility to binding by transcription factors. In contrast, histone deacetylation by histone deacetylase (HDAC) represses gene transcription by promoting DNA winding, thereby limiting access to transcription factors (11,12). In patients with severe asthma, several molecular mechanisms, including reduced HDAC2 expression, have been identified that might account for reduced steroid responsiveness (8). A recent study showed that total HDAC2 activity correlated negatively with the inhibitory effect of dexamethasone (Dx) on tumor necrosis factor (TNF)-α-induced interleukin (IL)-8 production in alveolar macrophages from smokers and nonsmokers (13). Theophylline might restore GC sensitivity via enhancement of HDAC2 activity in COPD macrophages (14). HDAC2 does not interact directly with NF-κB but may regulate NF-κB activity through its association with HDAC1 (15) or GR deacetylation to enable NF-κB suppression (9). Strategies for managing steroid resistance include the use of alternative anti-inflammatory drugs. In addition, a novel approach to reverse steroid resistance is to increase HDAC2 expression, which can be achieved by theophylline and phosphoinositide 3-kinase inhibitors (16,17). Reversal of corticosteroid resistance in COPD patients by restoring HDAC2 levels was shown to be effective in a small study (14,18), but long-term studies are needed to determine whether novel HDAC2 activators or theophylline might delay disease progression or reduce exacerbations or mortality.

1,25-Dihydroxyvitamin D_3_ (1,25(OH)_2_D_3_), the biologically active metabolite of vitamin D_3_, is a secosteroid hormone known to be involved in mineral and skeletal homeostasis. The discovery of 1,25(OH)_2_D_3_ receptor expression in lymphocytes and monocytes (19) suggested an additional role for this hormone in the immune system. Epidemiologic data have suggested that low serum vitamin D levels in children with asthma are associated with exacerbations, reduced lung function, and increased medication usage (20). *In vitro* studies have demonstrated that vitamin D enhances steroid responsiveness in regulatory T cells in adult asthmatics (21). Vitamin D may play an important role in pulmonary health by inhibiting inflammation, in part through maintaining regulatory T cells and direct induction of innate antimicrobial immunity (22-24). We have previously demonstrated that administration of 1,25(OH)_2_D_3_ improved the host response to inflammation and reduced the expression of inducible nitric oxide synthase (iNOS) in a rat asthma model. We suggested that 1,25(OH)_2_D_3_ might be a novel antioxidant drug and therapeutic agent in the treatment of asthma (25). However, little is known about the molecular mechanisms by which 1,25(OH)_2_D_3_ affects asthma pathogenesis. The current study aimed to determine whether 1,25(OH)_2_D_3_ administration could alter the expression and activity of HDAC2, which is involved in GC-dependent repression of NF-κB-induced gene expression, and whether combinations of vitamin D with corticosteroids may have additional benefits in regulating HDAC2 expression.

## Material and Methods

### Induction of allergic asthma and experimental design

Male Wistar rats (5 weeks old) with an average weight of 200±30 g were provided by the SLRC Experimental Animal Company (China). A modified protocol of immunization with ovalbumin (OVA; Sigma, USA) was used to induce allergic asthma in rats (25). Briefly, on day 0, animals received a subcutaneous injection of 1 mg OVA plus aluminum hydroxide (200 mg/mL in 0.9% NaCl, Sigma) and a 1-mL intraperitoneal injection of heat-killed *Bordetella pertussis* bacteria (6×10^9^; Shanghai Institute of Biological Products, China). On day 7, an intraperitoneal injection of OVA with aluminum hydroxide was performed. Rats in the negative control group were injected with 1 mL saline containing 200 mg/mL aluminum hydroxide. Two weeks later, the rats were placed unrestrained in a transparent plastic chamber (with an approximate volume of 4 L) connected to a nebulizer (Type 37.00, PARI BOY, Germany), and subjected to bronchial allergen challenge by inhalation of OVA (10 mg/mL saline) for 20 min. The challenge was carried out once a day for 6 consecutive days. The animals in the negative control group were challenged with saline.

Animals were randomly divided into six groups. 1) Negative control (-/-, n=5): no sensitization and no treatment. The animals only received nut oil, which is the solvent for 1,25(OH)_2_D_3_ (Roche, USA). 2) Positive control (OVA/OVA, n=5): sensitization and challenge with OVA. 3) 1,25(OH)_2_D_3_ pretreatment (OVA/OHD_3_ Ptr, n=5): sensitization and subsequent challenging with OVA; each rat was also given oral 1,25(OH)_2_D_3_ at 0.25 μg/day (26) by lavage throughout the experiment (from day 0 to 20). 4) 1,25(OH)_2_D_3_-treated group (OVA/OHD_3_ T, n=5): sensitization and challenging with OVA; the rats in this group were given oral 1,25(OH)_2_D_3_ at 0.25 μg/day 1 h before each challenge (from day 15 to 20). 5) Dx-treated group (OVA/Dx, n=5): sensitization and later challenging with OVA. The rats were treated with subcutaneous injection of Dx (300 µg) 1 h before each challenge (from day 15 to day 20). 6) Dx and 1,25(OH)_2_D_3_-treated group (OVA/OHD3+Dx, n=5). In addition to sensitization and later challenging with OVA, animals were treated with a subcutaneous injection of Dx (300 µg) and oral administration of 1,25(OH)_2_D_3_ (0.25 μg/day) before each challenge (from day 15 to 20).

All animals were housed 5 rats per cage under environmentally controlled conditions in compliance with the Shanghai Jiaotong University Policy on Animal Care and Use. All experiments were carried out with the approval of the Ethics Committee of the Faculty of Pharmacy, Shanghai Jiaotong University.

### BAL fluid collection and determination of cell numbers

Animals were euthanized with an overdose of pentobarbital 24 h after the last OVA exposure. A catheter was inserted into the trachea, and bronchoalveolar lavage (BAL) was performed using 10 mL saline. The BAL fluid was centrifuged at 250 *g* for 10 min at 4°C, the cell pellet was resuspended in 1 mL saline, and total cell counts were carried out. To perform the differential leukocyte cell count, 0.1 mL of the cell suspension was smeared on a glass slide and stained with Wright-Giemsa solution. Four hundred nucleated cells were then examined under a microscope.

### Histological analysis of lung tissues

After BAL, the right lobes of the lungs of each animal were removed for histological evaluation. The tissue was immediately immersed in Bouin's fixative for 48 h before it was embedded in paraffin. Paraffin-embedded tissue was sectioned at a thickness of 4 μm and stained with hematoxylin-eosin. Photodocumentation was prepared with a Zeiss microscope (Carl Zeiss Shanghai Co., Ltd., China) and analyzed with the Image-Pro Plus software (USA). The inflammatory cell infiltration in the lung was evaluated with a 10-point scoring system. In brief, one section per lung was individually assessed in three categories: peribronchial/peribronchiolar inflammation, perivascular inflammation, and alveolar inflammation. Peribronchial/peribronchiolar and perivascular inflammation were individually scored from 0-4 (0, none; 1, thin inflammatory infiltrate [<3 cell layers] confined to the central lung; 2, dense inflammatory infiltrate [≥3 cell layers] confined to the central lung; 3, thin [<3 cell layers] to dense [≥3 cell layers] inflammatory infiltrate extending to the peripheral airways/vessels; 4, dense [≥3 cell layers] inflammatory infiltrate extending to the pleural surface). Alveolar inflammation was scored from 0 to 2 (0, absent; 1, few foci present; 2, many foci present). Each lung section was given an overall score in each of the three scoring categories. The scores were then summed to give a total inflammatory score (maximum score of 10).

### Cytokine production in BAL

The concentrations of cytokines and chemokines in BAL specimens were measured with an enzyme-linked immunosorbent assay (ELISA; R&D Systems, USA). The limits of detection were TNF-α, 20 pg/mL; IL-8, 50 pg/mL; IL-5, 2.0 pg/mL; IL-13, 2.5 pg/mL; and granulocyte-macrophage colony-stimulating factor (GM-CSF), 40 pg/mL. All assays were performed in duplicate, and the mean values were used for statistical analysis.

### Quantitative reverse transcriptase-polymerase chain reaction (qRT-PCR)

Total RNA was extracted from lung tissues with TRIzol reagent (Invitrogen, USA) according to the manufacturer's protocol. Reverse transcription (RT) was performed with oligo (dT) primer using the RevertAid^™^ First Strand cDNA Synthesis Kit (Fermentas, China). PCR primer sequences included the following: HDAC2, 5′-CGGTGGCTCAGTTGCTGGGG-3′ (sense) and 5′-TGCAGTCCTCCCGCCCAGTT-3′ (antisense); NF-κB p65, 5′-GACGAGGCTCGGAGAGCCCA-3′ (sense) and 5′-CTGGGGCGGCTGACCGAATG-3′ (antisense); and glyceraldehyde 3-phosphate dehydrogenase (GAPDH), 5′-CAAGTTCAACGGCACAGTCAAGG-3′ (sense) and 5′-ACATACTCAGCACCAGCATCACC-3′ (antisense). The predicted sizes of the PCR products were 78 bp for HDAC2, 145 bp for NF-κB p65, and 123 bp for GAPDH. The 50-µL PCR reactions consisted of 0.5 µL of each primer, 32.5 µL SYBRGreen Mix, 14.5 µL ddH_2_O, and 2 µL cDNA. qRT-PCR was performed in triplicate with a Mastercycler ep gradient S thermocycler (Eppendorf, Germany). The resulting product of each sample was normalized to that of GAPDH transcripts. The quantitative analysis was carried out using the ΔΔCT method.

### Western blot analysis of HDAC2

Freshly removed lung tissue was homogenized in lysis buffer (50 mM Tris [pH 7.4], 150 mM NaCl, 1% Triton X-100, and 1 mM EDTA) and centrifuged at 14,000 *g* for 5 min. The supernatant containing 40 μg of protein was mixed with 10 µL sample buffer (125 mM Tris-HCl [pH 6.8], 4% SDS, 3.5 mM DTT, 0.02% bromophenol blue, and 20% glycerol), boiled for 2-3 min, and loaded onto an 8% SDS-PAGE gel with a protein molecular mass marker. Separated proteins in the gel were transferred to a PVDF membrane (Polyscreen NEF 1000, NEN Life Science Products, USA) using a blot transfer system (Bio-Rad Laboratories, USA). After blocking with 5% BSA in Tris-buffered saline (TBS) solution (20 mM Tris and 500 mM NaCl, pH 7.5) overnight at 4°C, membranes were incubated with a polyclonal rabbit anti-HDAC2 antibody (1:1000, Abcam, UK) and a polyclonal anti-NF-κB p65 antibody (1:1000, Cell Signaling Technology, USA) at room temperature for 1 h. After washing three times with TBS with Tween (0.1% Tween 20, 100 mM Tris-HCl, and 150 mM NaCl, pH 7.5), the membrane was incubated with a goat anti-rabbit IgG antibody conjugated with horseradish peroxidase (HRP) at room temperature for 1 h. The membrane was washed again and developed with a Vectastain ABC detection kit (Vector Labs, USA). β-Actin was probed with an anti-actin antibody (1:1000, Santa Cruz Biotechnology, USA) as an internal control. Densitometry was performed using ImageJ software from the US National Institutes of Health (http://rsb.info.nih.gov/ij/).

### Immunohistochemical detection of HDAC2 in lung cells

The presence of HDAC2 in lung cells was analyzed by immunohistochemistry. Tissue sections were deparaffinized, treated with H_2_O_2_, and blocked with 5% normal rabbit serum in phosphate-buffered saline (PBS). After washing with PBS, the tissue sections were incubated with a rabbit anti-HDAC2 antibody (1:1000, Santa CruzBiotechnology) overnight at 4°C. Following rinsing with PBS, the sections were incubated with biotinylated anti-rabbit IgG for 2 h. Specific binding was detected with an avidin-biotin-HRP complex and diaminobenzidine (DAB) kit (Vector Labs). The slides were then counterstained with hematoxylin, dehydrated through graded alcohol and xylenes, and mounted on coverslips. The sections were examined with an LSM 5 PASCAL confocal microscope (Carl Zeiss Shanghai Co., Ltd.). Each slide was rated according to the ratio of HDAC2-positive cells to all the cells in the staining area. The immunohistochemistry results were evaluated independently by two investigators in a blinded manner. The percentages of HDAC2-expressing cells were determined by counting at least 600 cells in 3 or more representative microscopic areas.

### Immunoprecipitation and enzyme activity assay of HDAC2

A 200-μg aliquot of protein from lung tissue in 100 µL was incubated for 1 h with anti-HDAC2 antibody (1:1000; Abcam) before protein A/G agarose beads (40 µL, Santa Cruz Biotechnology) were added for further incubation at 4°C overnight with constant agitation. HDAC2 activity was measured with a colorimetric assay kit (Biomol, USA) in which HDAC substrate will produce a chromophore after being deacetylated. HeLa cell nuclear extract was used as a positive control. A standard curve was prepared using the indicated amount of the deacetylated standard (Boc-Lys-pNA) included in the kit. The results are reported as micromolar values of the provided standard per milligram of protein.

### Statistical analysis

Cell numbers in BAL and NF-κB p65 and HDAC2 mRNA levels are reported as means±SD. Mann-Whitney *U* tests were used to analyze lung inflammation scores. The data were analyzed using SPSS for Windows version 15 (SPSS Institute, Inc., USA). Analyses of variance (ANOVA) and Student-Newman-Keuls tests were used to compare data from multiple groups, and P<0.05 was considered to be statistically significant.

## Results

### Administration of 1,25(OH)_2_D_3_ reduced total cells and eosinophils in BAL fluid

**Figure 1:**
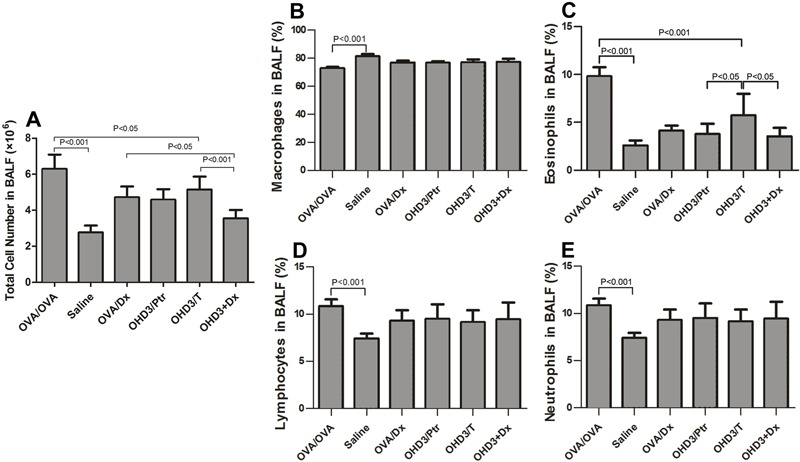
Effects of different treatments on the number of total and differential cells in bronchoalveolar lavage fluid (BALF). Data are reported as means±SD (n=5/group). *A*, The total cells in the BALF were significantly higher in the OVA/OVA group than in the control group (P<0.001). In the OHD/3 Ptr group, as well as the OHD3/T, OVA/Dx or OHD3+Dx groups, the total BALF cells were decreased compared to those in the OVA/OVA group (P<0.05). The total cell number was also lower in the OHD3+Dx group than in the OHD3/T or OVA/Dx groups (P<0.05). *B*-*E*, There was a significantly higher percentage of eosinophils and lymphocytes in BALF, but a decrease in the percentage of macrophages in the OVA/OVA group compared with the saline control animals (P<0.001). The proportion of eosinophils in the BALF from OHD3/Ptr, and OHD3/T and OHD3+Dx groups was less than that from the OVA/OVA group (P<0.001, *C*). The eosinophil percentage was significantly lower in the OHD3+Dx group than in the OHD3/T group (P<0.05). There was a slight but significant decrease in the eosinophil percentage in the OHD3/Ptr group than in the OHD3/T group (P<0.05, *C*). ANOVA and Student-Newman-Keuls tests were used for statistical analyses. OHD3/Ptr: 1,25(OH)_2_D_3_ pretreatment group; OHD3/T: 1,25(OH)_2_D_3_ treated group; OVA/OVA: asthma group; OVA/Dx: dexamethasone treated group; saline: the control group; OHD3+Dx: dexamethasone and 1,25(OH)_2_D_3_treated group.

To assess the effects of 1,25(OH)_2_D_3_ on the development of allergic inflammation following OVA challenge in rats, the numbers of cells in BAL fluid were counted. Strikingly, the number of total cells in BAL fluid collected from the OVA/OVA rats was greater than those from the negative control animals (6.30±0.79×10^6^ *vs* 2.77±0.38×10^6^, P<0.001, Figure 1A). Pretreatment with 1,25(OH)_2_D_3_, as well as treatment with 1,25(OH)_2_D_3_, Dx, or both inhibited the OVA-induced increase of the total cell number in the BAL fluid (P<0.05, Figure 1A). The total cell number was also lower in the 1,25(OH)_2_D_3_ with Dx treatment group (3.54±0.48×10^6^) than in the 1,25(OH)_2_D_3_ alone (5.15±0.72×10^6^, P<0.001) or Dx alone treatment groups (4.72±0.60×10^6^, P<0.05, Figure 1A). The total cells in BAL were slightly decreased in the 1,25(OH)_2_D_3_-pretreated group compared to 1,25(OH)_2_D_3_-treated animals, although the difference was not statistically significant (P>0.05, Figure 1A).

The differential cell counting results revealed that the majority of cells in BAL fluid were macrophages, and no significant difference was observed among the treatment groups (P>0.05, Figure 1B). However, significantly higher percentages of eosinophils and lymphocytes were noted in the OVA/OVA animals than in the negative control animals (P<0.001, Figure 1C and D). The proportion of eosinophils in BAL fluid from the 1,25(OH)_2_D_3_pretreatment, 1,25(OH)_2_D_3_, or 1,25(OH)_2_D_3_ plus Dx-treated rats were 3.8±1.05, 5.75±2.24, and 3.55±0.87%, respectively, compared to 9.8±0.96% in the OVA/OVA group (P<0.001, Figure 1C). The eosinophil percentage in BAL was significantly lower in the 1,25(OH)_2_D_3_ combined Dx-treated group than in the 1,25(OH)_2_D_3_-treated group (P<0.05, Figure 1C). There was a slight but significant decrease in eosinophils in the 1,25(OH)_2_D_3_pretreatment group compared to the 1,25(OH)_2_D_3_ treatment group (P<0.05, Figure 1C). However, there were no significant differences in eosinophil percentages between the Dx-treated and the 1,25(OH)_2_D_3_ plus Dx treatment groups (P>0.05, Figure 1C). No significant differences were observed in the percentages of lymphocytes and neutrophils among the treatment groups (P>0.05, Figure 1D and E).

### 1,25(OH)_2_D_3_ treatment reduced lung inflammation

**Figure 2:**
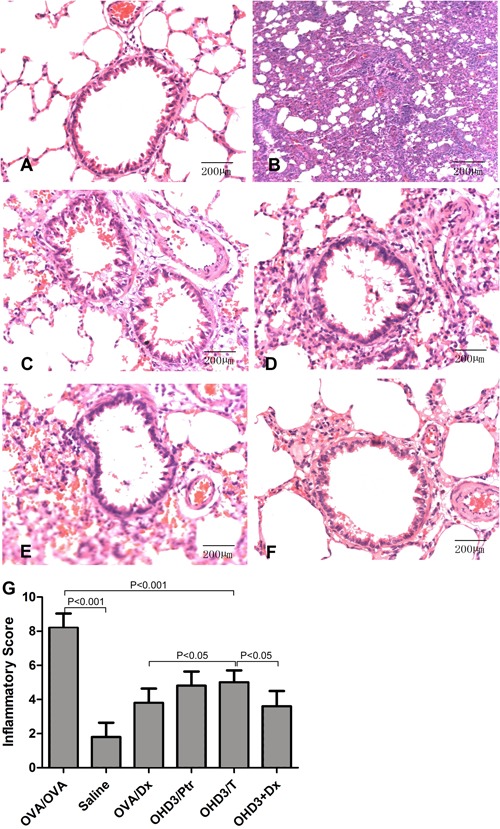
Representative images of paraffin-embedded and hematoxylin-eosin-stained sections of the right lobe of lungs from rats treated with saline (*A*), OVA/OVA (*B*), OVA/Dx (*C*), OHD3/Ptr (*D*), OHD3/T (*E*), OHD3+Dx (*F*). *G*, Effect of different treatments on the inflammation score. Mann-Whitney *U* tests were used for statistical analyses. Saline: control group; OVA/OVA: asthma group; OVA/Dx: dexamethasone treated group; OHD3/Ptr: 1,25(OH)_2_D_3_ pretreatment group; OHD3/T: 1,25(OH)_2_D_3_ treated group; OHD3+Dx: dexamethasone and 1,25(OH)_2_D_3_ treated group.

In negative control animals, the small bronchi, bronchioles, and lung alveoli were structurally normal; the mucosal epithelia were intact; and no inflammation was observed (Figure 2A). In contrast, remarkable inflammatory changes were noted in the airways of the OVA/OVA rats, including desquamation of the bronchial epithelia; the presence of secretion fluid and damaged cells inside the bronchi and alveoli lumina; and patchy inflammatory infiltrations in the bronchial submucosa, perivascular areas, and the surrounding alveolar septa. The infiltrates consisted primarily of mononuclear cells and some eosinophils. We also observed that OVA exposure induced goblet cell hyperplasia, hemorrhage, congestion, and alveolar and interstitial edema (Figure 2B). Notably, the 1,25(OH)_2_D_3_ pretreatment and treated groups exhibited less inflammatory cell infiltration in peribronchial and perialveolar areas, decreased interstitial edema, and fewer epithelial lesions in the bronchi and bronchioles. The goblet cell hyperplasia and congestion triggered by OVA exposure also appeared to be affected by 1,25(OH)_2_D_3_treatment (Figure 2C-2F). The inflammatory score in the OVA/OVA rats was significantly higher than that in the control animals (8.2±0.83 *vs* 1.8±0.84, P<0.001; Figure 2G). Both 1,25(OH)_2_D_3_ pretreatment and treatment significantly reduced the inflammatory score in the OVA/OVA rats (P<0.05, Figure 2G). The inflammatory score was also reduced in the 1,25(OH)_2_D_3_ plus Dx treatment group compared to the 1,25(OH)_2_D_3_-treated and the Dx-treated groups (P<0.05, Figure 2G).

### 1,25(OH)_2_D_3_ pretreatment and treatment reduced cytokine levels in BAL

**Figure 3:**
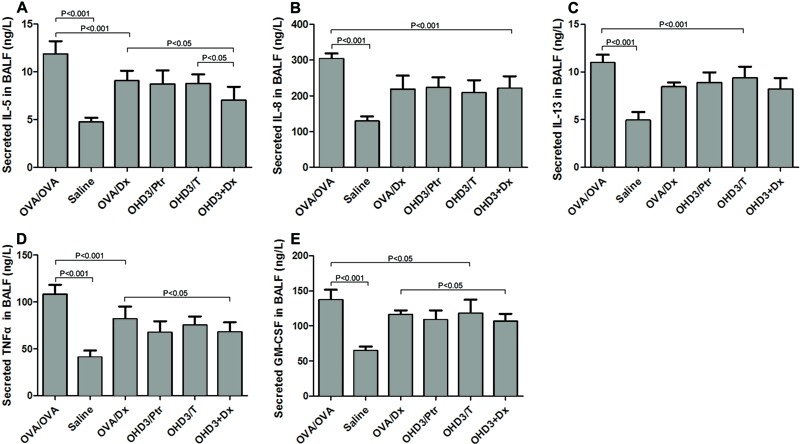
Effects of different treatments on the cytokine release in the bronchoalveolar lavage fluid (BALF). Data are reported as means±SD (n=5/group). The concentrations of all cytokines tested were significantly higher in the OVA/OVA group than in the control group (P<0.05). The 1,25(OH)_2_D_3_ pretreatment and treatment significantly decreased the concentrations of TNF-α, IL-5, IL-13, IL-8 and GM-CSF in the BALF compared to those in the OVA/OVA group (P<0.05). Among the cytokines measured, the concentrations of IL-5, GM-CSF, TNF-α were lower in the OHD3+Dx group than those in OVA/Dx (P<0.05). ANOVA and Student-Newman-Keuls tests were used for statistical analyses. Saline: control group; OVA/OVA: asthma group; OVA/Dx: dexamethasone treated group; OHD3/Ptr: 1,25(OH)_2_D_3_ pretreatment group; OHD3/T: 1,25(OH)_2_D_3_ treated group; OHD3+Dx: dexamethasone and 1,25(OH)_2_D_3_ treated group; TNF-α: tumor necrosis factor-α; IL: interleukin; GM-CSF: granulocyte-macrophage colony-stimulating factor.

To investigate the relationship between HDAC2 activity and inflammatory gene expression levels in OVA/OVA rats, cytokine levels in the BAL samples were analyzed by ELISA. The concentrations of all tested cytokines (TNF-α, IL-5, IL-13, IL-8, and GM-CSF) were significantly higher in the OVA/OVA rats than in the negative controls (P<0.05, Figure 3). Both 1,25(OH)_2_D_3_ pretreatment and treatment significantly reduced the BAL fluid concentrations of all tested cytokines compared to levels in the OVA/OVA rats (P<0.05, Figure 3). The combined treatment of 1,25(OH)_2_D_3_ and Dx also reduced the expression levels of cytokines compared to the OVA/OVA group (P<0.05, Figure 3). Among the cytokines measured, the concentrations of IL-5, GM-CSF, and TNF-α but not IL-13 and IL-8 were lower in the 1,25(OH)_2_D_3_ and Dx-treated rats than in those treated only with Dx (P<0.05, Figure 3). In contrast, there were no significant differences in cytokine levels between the 1,25(OH)_2_D_3_ pretreatment and treatment groups (P>0.05, Figure 3).

### NF-κB p65 and HDAC2 mRNA expression in pulmonary tissues

**Figure 4:**
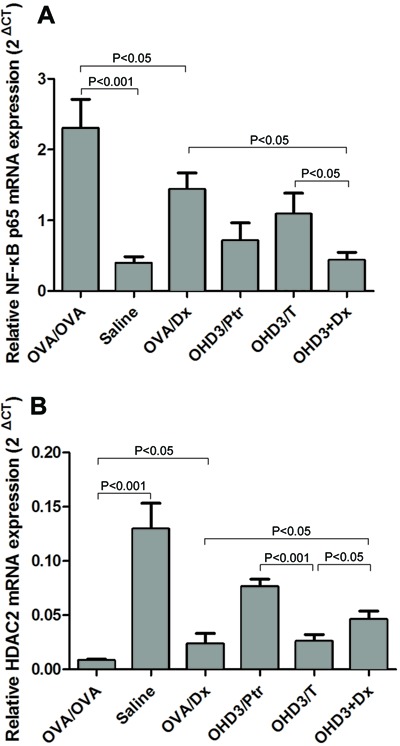
Effects of different treatments on NF-κB p65 and HDAC2 gene transcription in the lung. Bars represent mean±SD (n=5/group). The relative ratio of mRNA of NF-κB p65 to that of GAPDH was significantly higher in the OVA/OVA group than in the control group (P<0.001, *A*). 1,25(OH)_2_D_3_pretreatment, treatment and the combined treatment significantly decreased NF-κB p65 mRNA expression in the OVA sensitized and challenged rats (P<0.001). The NF-κB p65 mRNA level in OHD3+Dx was also lower than that in the OVA/Dx and OHD3/T groups (P<0.05). In contrast, the relative ratio of mRNA of HDAC2 to that of GAPDH was significantly lower in the OVA/OVA group than in the control group (P<0.001). 1,25(OH)_2_D_3_pretreatment, treatment and the combined treatment significantly increased the expression level of HDAC2 mRNA in the OVA sensitized and challenged rats (P<0.001). The HDAC2 mRNA level was higher in the OHD3/Ptr group than in the OHD3/T group (P<0.001). The HDAC2 mRNA level in the OHD3+Dx group was also higher than that in the OVA/Dx and OHD3/T groups (P<0.05). ANOVA and Student-Newman-Keuls tests were used for statistical analyses. Saline: control group; OVA/OVA: asthma group; OVA/Dx: dexamethasone treated group; OHD3/Ptr: 1,25(OH)_2_D_3_ pretreatment group; OHD3/T: 1,25(OH)_2_D_3_ treated group; OHD3+Dx: dexamethasone and 1,25(OH)_2_D_3_ treated group.

To study NF-κB p65 and HDAC2 gene expression in lung tissues, NF-κB p65 and HDAC2 mRNA levels were measured with qRT-PCR. As shown in Figure 4A, NF-κB p65 mRNA expression level was significantly higher in the OVA/OVA rats than in the negative control animals (2.30±1.01 *vs* 0.39±0.09, P<0.001). Both 1,25(OH)_2_D_3_ pretreatment and treatment significantly reduced NF-κB p65 mRNA expression in the OVA/OVA rats (0.71±0.25, P<0.001 and 1.09±0.30, P<0.001, respectively). The combined treatment of 1,25(OH)_2_D_3_ and Dx reduced NF-κB p65 gene expression compared to the OVA/OVA group (P<0.001), as well as the Dx-treated and 1,25(OH)_2_D_3_-treated groups (P<0.05).

In contrast, HDAC2 mRNA expression was significantly lower in the OVA/OVA rats than in the negative controls (0.008±0.001 *vs*0.13±0.02, P<0.001; Figure 4B). Both 1,25(OH)_2_D_3_ pretreatment and treatment significantly increased HDAC2 mRNA expression in the OVA/OVA rats (0.076±0.007, P<0.001 and 0.026±0.006, P<0.001, respectively). The HDAC2 mRNA level was higher in the 1,25(OH)_2_D_3_ pretreated group compared to the 1,25(OH)_2_D_3_-treated group (P<0.001). The combined treatment of 1,25(OH)_2_D_3_ and Dx also increased HDAC2 expression compared to that in the OVA/OVA group (P<0.001, Figure 4B) and the Dx-treated and 1,25(OH)_2_D_3_-treated groups (P<0.05, Figure 4B).

### NF-κB p65 and HDAC2 protein expression in pulmonary tissues

**Figure 5:**
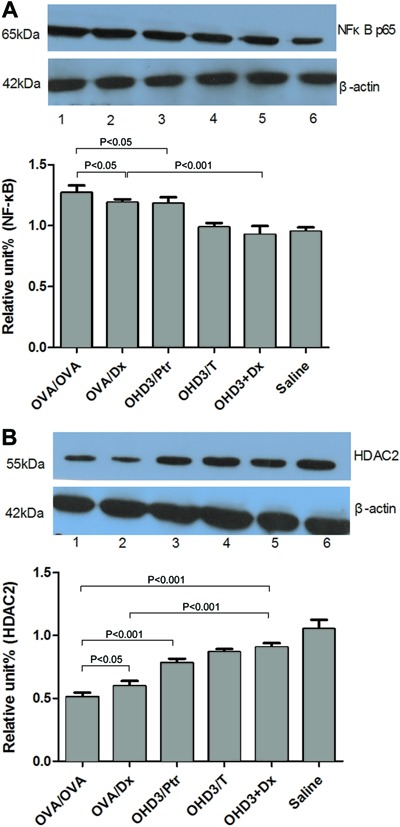
Effect of different treatments on nuclear factor kappa B (NF-κB) p65 and histone deacetylase 2 (HDAC2) protein expression in the lung tissues. *A*, Effect of different treatments on NF-κB p65 protein expression. A representative Western blot is shown in the upper panel and the analysis of the ratio between NF-κB and β-actin by densitometry is shown in the lower panel. The relative NF-κB p65 protein levels were higher in the OVA/OVA group than that in the saline controls (P<0.001), but such increase was significantly attenuated in rats pretreated or treated with 1,25(OH)_2_D_3_ (P<0.05). The combined treatment with 1,25(OH)_2_D_3_ and dexamethasone further decreased NF-κB p65 to that of the saline control group (P<0.001; *A*). *B*, Effect of different treatments on HDAC2 protein expression in the lung tissues. A representative Western blot is shown in the upper panel and the analysis of the ratio between HDAC2 and β-actin by densitomitry is shown in the lower panel. The relative HDAC2 protein levels were decreased significantly in the OVA/OVA group compared to the saline controls (P<0.001), but such decrease was decreased significantly in the animals pretreated or treated with 1,25(OH)_2_D_3_(P<0.05). *Lanes* in Western blots: *1*, OVA/OVA (asthma group); *2*, OVA/Dx (dexamethasone treated group); *3*, OHD3/Ptr (1,25(OH)_2_D_3_ pretreatment group); *4*, OHD3/T (1,25(OH)_2_D_3_treated group); *5*, OHD3+Dx (dexamethasone and 1,25(OH)_2_D_3_ treated group); *6*, saline (negative control group). ANOVA and Student-Newman-Keuls tests were used for statistical analyses.

Western blot analysis was employed to semi-quantitatively determine protein expression levels of NF-κB p65 (65 kDa), HDAC2 (55 kDa), and β-actin (42 kDa) (Figure 5). The relative NF-κB p65 protein levels (ratios of NF-κB p65 to β-actin) analyzed by densitometry were higher in the OVA/OVA rats than in the negative control animals (P<0.001, Figure 5A). However, this increase was significantly attenuated in rats pretreated or treated with 1,25(OH)_2_D_3_ (P<0.05, Figure 5A). The combined treatment with 1,25(OH)_2_D_3_ and Dx resulted in NF-κB p65 expression comparable to that in the negative control group (Figure 5A). The relative NF-κB p65 protein level in this group was also lower than that in the Dx-treated group (P<0.001, Figure 5A).

In contrast to the increasing expression of NF-κB p65, the relative protein level of HDAC2 was decreased in the OVA/OVA rats compared to the negative control group (P<0.001, Figure 5B). Interestingly, rats pretreated and treated with 1,25(OH)_2_D_3_ showed increased HDAC2 protein levels (P<0.001, Figure 5B). The combined treatment of 1,25(OH)_2_D_3_ and Dx significantly increased the HDAC2 protein level to nearly that of the negative control group. The ratio in this group was also higher than that in the Dx-treated group (P<0.001, Figure 5B).

### Immunohistochemical examination of HDAC2

**Figure 6:**
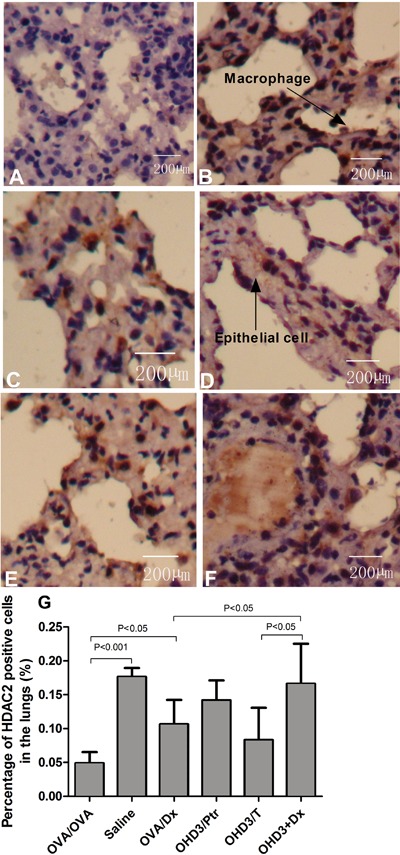
Immunohistochemical study of the localization of histone deacetylase 2 (HDAC2) in the lungs. *A*, OVA/OVA; *B*, saline; *C*, OVA/Dx; *D*, OHD3/Ptr; *E*, OHD3/T; *F*, OHD3+Dx; *G*, effect of different treatments on the percentage of HDAC2 positive cells in the lungs (%). ANOVA and Student-Newman-Keuls tests were used for statistical analyses. OHD3/Ptr: 1,25(OH)_2_D_3_ pretreatment group; OHD3/T: 1,25(OH)_2_D_3_ treated group; OVA/OVA: asthma group; OVA/Dx: dexamethasone treated group; saline: the control group; OHD3+Dx: dexamethasone and 1,25(OH)_2_D_3_treated group.

HDAC2 immunoreactivity was detected primarily in the nuclei of the epithelial cells and macrophages at the apical region of the airway in the rat lung (Figure 6A-F). The percentage of HDAC2-positive cells was calculated (Figure 6G) and was found to be higher in the pretreatment and treatment groups than in the OVA/OVA group (P<0.05, Figure 6G). The combined treatment of 1,25(OH)_2_D_3_ and Dx significantly increased the number of HDAC2-positive cells in the lungs compared to OVA/OVA rats (Figure 6G), as well as to those in the Dx-treated and 1,25(OH)_2_D_3_-treated groups (P<0.05, Figure 5B).

### Effect of 1,25(OH)_2_D_3_ and Dx on HDAC2 enzymatic activity

**Figure 7:**
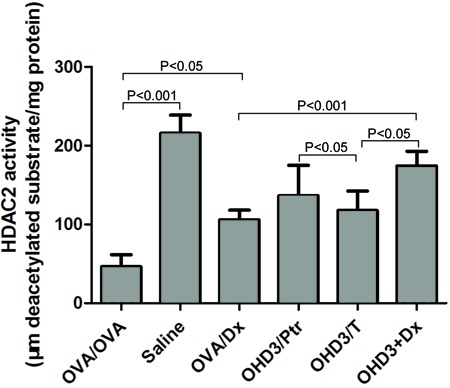
Effect of 1,25(OH)_2_D_3_ and dexamethasone on histone deacetylase 2 (HDAC2) activity measured by a colorimetric assay kit. HeLa cell nuclear extract was used as a positive control. The activity of HDAC2 was decreased in the OVA/OVA group compared to the control group (P<0.05); both pretreatment and treatment with 1,25(OH)_2_D_3_ increased HDAC2 activity (P<0.05). The combined treatment of 1,25(OH)_2_D_3_ and dexamethasone increased HDAC2 activity compared to the OVA/OVA group and OVA/Dx groups (P<0.001). ANOVA and Student-Newman-Keuls tests were used for statistical analyses. OHD3/Ptr: 1,25(OH)_2_D_3_ pretreatment group; OHD3/T: 1,25(OH)_2_D_3_ treated group; OVA/OVA: asthma group; OVA/Dx: dexamethasone treated group; saline: the control group; OHD3+Dx: dexamethasone and 1,25(OH)_2_D_3_treated group.

We biochemically measured HDAC2 enzyme activity in the proteins immunoprecipitated with HDAC2 antibody and protein A/G agarose beads from lung homogenate with a colorimetric kit. HDAC2 activity in the lung of the OVA/OVA rats was decreased compared to that in the negative control animals (P<0.001, Figure 7). However, pretreatment with 1,25(OH)_2_D_3_, treatment with 1,25(OH)_2_D_3_ or Dx, or a combination of 1,25(OH)_2_D_3_ and Dx were all able to increase HDAC2 activity (P<0.05, Figure 7). The combined treatment of 1,25(OH)_2_D_3_ and Dx further increased HDAC2 activity compared to that of the 1,25(OH)_2_D_3_ or Dx alone groups (P<0.05, Figure 7). In addition, HDAC2 activity was higher in the 1,25(OH)_2_D_3_pretreatment group than in the 1,25(OH)_2_D_3_-treated group (P<0.05, Figure 7).

## Discussion

A growing body of scientific and medical literature supports the theory that vitamin D has anti-inflammatory functions in health and disease in addition to its roles in calcium metabolism and bone health (26). Recent publications have shown that asthmatics with low serum vitamin D have impaired lung function, increased airway hyper-reactivity, and increased corticosteroid dependence (22,23,27). GC application appears to be independently associated with vitamin D deficiency, and a need for screening and repletion of vitamin D in patients on chronic steroids was suggested (27). Other studies proposed that vitamin D may enhance GC responsiveness (28). Although the mechanism for such enhancement has not been elucidated, HDAC2 might mediate the effects of steroids by switching off activated inflammatory genes (29). HDAC2 expression and enzyme activity are reduced by oxidative stress in patients with COPD, severe asthma, and in smokers with asthma (8,9,29-32). The aim of current study was to investigate whether 1,25(OH)_2_D_3_ administration would alter the expression of HDAC2, which is involved in the GC-dependent repression of NF-κB-induced gene expression, and whether increased HDAC2 expression could enhance GC responsiveness in GC-insensitive diseases such as an animal model of asthma.

We induced asthma in rats by OVA sensitization/challenging and observed that the total number of cells in BAL fluid from these animals was significantly higher than those from negative control rats, confirming that lung asthma was successfully induced (25). Recent investigations (3,4,33) have shown that asthma induces inflammatory cytokine expression via an NF-κB-dependent pathway. In the current study, gene expression levels of chemotactic factors including IL-8, IL-5, IL-13, TNF-α, and GM-CSF, all of which are NF-κB mediated, were increased in BAL from OVA/OVA rats compared to the control group. NF-κB p65 mRNA and protein levels were also elevated in the OVA/OVA group. Consistent with previous reports that HDAC2 expression was reduced by oxidative stress in patients with COPD and severe asthma (8,9,29-32), our results showed that HDAC2 gene expression was significantly lower in OVA/OVA rats than in saline controls. We demonstrated an increase in the release of the cytokines GM-CSF, TNF-α, and IL-8 in experimental asthma. This effect might result in enhanced acetylation and local DNA unwinding, which could cause increased inflammatory gene expression.

In order to study the potential therapeutic effect of 1,25(OH)_2_D_3_ on allergic asthma, it was administered orally on day 1 and throughout the experiment (pretreatment), or before each OVA challenge (treatment). The data suggested that both pretreatment and treatment downregulated the inflammatory response, as demonstrated by the histological examination of lung tissue and the total and differential cell counts in BAL fluid. 1,25(OH)_2_D_3_ appeared to have similar effects to Dx, which is an effective drug for asthma control. Both 1,25(OH)_2_D_3_pretreatment and treatment significantly increased HDAC2 gene expression and reduced NF-κB p65 mRNA and protein levels in the OVA/OVA group. Immunohistochemical staining for HDAC2 in the lung tissue showed that its expression was limited to macrophages and epithelial cells as previously described (13,14,34), and importantly, the percentage of HDAC2-positive cells was higher in the pretreatment and treatment groups than in the OVA/OVA group. It was reported that oxidative and nitrative stresses induce the rapid formation of peroxynitrite, which is increased in exhaled breath condensate from patients with COPD and asthma (17). Peroxynitrite nitrates affect select tyrosine residues on certain proteins. For example, HDAC2, but not other isoforms of HDAC, shows increased tyrosine nitration in macrophages and peripheral lung in patients with COPD and asthma (34). Nitration of HDAC2 inactivates the enzyme's catalytic activity and also leads to its ubiquitination, which marks it for degradation by the proteasome, resulting in decreased HDAC2 protein levels in the lungs of patients with severe COPD and asthma (31). We previously demonstrated that administration of 1,25(OH)_2_D_3_ eased the symptoms of inflammatory responses and reduced iNOS expression in a rat asthma model (25). It was also shown that 1,25(OH)_2_D_3_ exerts antioxidative effects by reducing iNOS expression and activity. Because the reduction of HDAC2 in patients with asthma could be due to inactivation of the enzyme by oxidative and nitrative stress (10,35), we speculate that 1,25(OH)_2_D_3_ might inhibit iNOS expression and activity, which would explain the increased HDAC2 expression levels in the 1,25(OH)_2_D_3_ pretreated and treated groups.

The percentage of eosinophils in BAL was decreased in the 1,25(OH)_2_D_3_ pretreatment group compared to the 1,25(OH)_2_D_3_ treatment group, but there was no difference in total cell numbers between the two groups. Although both the mRNA level and the enzyme activity of HDAC2 were higher in the 1,25(OH)_2_D_3_pretreatment group than in the 1,25(OH)_2_D_3_ treatment group, NF-κB p65 protein and mRNA levels and cytokine levels were not significantly different. Therefore, 1,25(OH)_2_D_3_ administration starting at the beginning of OVA sensitization may inhibit naïve T lymphocytes from skewing toward the Th2 phenotype, resulting in fewer eosinophils (36).

In this study, we observed synergistic effects of 1,25(OH)_2_D_3_and Dx on the suppression of total cell infusion in BAL; the total cell number in BAL fluid from the 1,25(OH)_2_D_3_ and Dx-treated group was lower than those in the groups treated with either 1,25(OH)_2_D_3_ or Dx. The combined treatment of 1,25(OH)_2_D_3_ and Dx also reduced eosinophil numbers and reduced expression levels of IL-5, GM-CSF, and TNF-α. This suggested that the decrease in total inflammatory cells in BAL fluid might be due to the suppression of cytokine gene expression in the 1,25(OH)_2_D_3_ and Dx-treated group because these cytokines are chemotactic to inflammatory cells, including macrophages and eosinophils (34). NF-κB p65 mRNA and protein expression levels were decreased in the 1,25(OH)_2_D_3_ and Dx-treated group compared to the OVA/OVA group, and they were also lower than those in the Dx-treated and 1,25(OH)_2_D_3_-treated groups. The combined treatment of 1,25(OH)_2_D_3_ and Dx increased the expression and activity of HDAC2 compared to the OVA/OVA and Dx-treated groups. GRs become acetylated after ligand binding, and HDAC2-mediated GR deacetylation enables GR binding to the NF-κB complex (9). The observed overexpression of HDAC2 in the combined treatment group might have partially restored GC sensitivity. Therefore, the suppression of NF-κB p65 expression and cytokine release in the lung could be due to increased expression of HDAC2 that inhibits NF-kB-activated gene expression (8,9).

Recent studies have shown that suppression of inflammatory genes by GC requires the recruitment of HDAC2 to the activation complex in the nucleus via GRs (37). This suggests that the decrease in HDAC2 expression may increase inflammatory gene expression and also reduce GC function. Vitamin D_3_ has been shown to reverse the defective induction of IL-10-secreting regulatory T cells in GC-resistant patients with asthma (21), which suggests that vitamin D_3_may have a therapeutic role in GC-resistant asthma. Our study showed that HDAC2 expression was significantly increased in the 1,25(OH)_2_D_3_- and Dx-treated rats but not in those treated with Dx alone. 1,25(OH)_2_D_3_ may increase GC function via its ability to increase HDAC2 levels. Therefore, 1,25(OH)_2_D_3_ and Dx may synergistically increase HDAC2 expression and enzyme activity.

In summary, HDAC2 gene expression and enzyme activity might be suppressed in patients with asthma, resulting in NF-κB activation and increased BAL levels of IL-8, IL-5, IL-13, GM-CSF, and TNF-α. Administration of 1,25(OH)_2_D_3_ could induce epigenetic modifications by increasing HDAC2, thereby reducing NF-κB activation and inflammatory cytokine release. Our results provide the first evidence that chromatin remodeling by 1,25(OH)_2_D_3_ may occur in the context of asthma. Future studies on chromatin alteration and its related molecular mechanism(s) are needed to understand the effect of this compound as a natural therapy for chronic inflammation associated with asthma.
